# Supplementary material for: Convergence and divergence in mortality: A global study from 1990 to 2030
Source: PLoS One. 2024 Jan 17;19(1):e0295842. doi: 10.1371/journal.pone.0295842 (PMC10793939; doi:10.1371/journal.pone.0295842)
Supplement: S1 Annex — (PDF) [file pone.0295842.s001.pdf]

A Annex 1

Table 1. List of the countries and dataset employed.

Country	Acronyms	Country	Acronyms	Country	Acronyms
Afghanistan	AFG	Ghana	GHA	Panama	PAN
Albania	ALB	Greece	GRC	Papua New Guinea	PN1
Algeria	DZA	Grenada	GRD	Paraguay	PRY
Angola	AGO	Guam	GUM	Peru	PER
Antigua and Barbuda	ATG	Guatemala	GTM	Philippines	PHL
Argentina	ARG	Guinea	GIN	Poland	POL
Armenia	ARM	Guinea-Bissau	GNB	Portugal	PR1
Aruba	ABW	Guyana	GUY	PuertoRico	PRI
Australia	AUS	Haiti	HTI	Qatar	QAT
Austria	AUT	Honduras	HND	Republic of Congo	COG
Azerbaijan	AZE	Hong Kong	HKG	Republic of Korea	KOR
Bahamas	BHS	Hungary	HUN	Romania	ROU
Bahrain	BHR	Iceland	ISL	Russian	RUS
Bangladesh	BGD	India	IND	Rwanda	RWA
Barbados	BRB	Indonesia	IDN	Saint Lucia	LCA
Belarus	BLR	Iran	IRN	Saint Vincent and Grenadines	VCT
Belgium	BEL	Iraq	IRQ	Samoa	WSM
Belize	BLZ	Ireland	IRL	São Tomé and Principe	STP
Benin	BEN	Israel	ISR	Saudi Arabia	SAU
Bhutan	BTN	Italy	ITA	Senegal	SEN
Bolivia	BOL	Jamaica	JAM	Serbia	SRB
Bosnia and Herzegovina	BIH	Japan	JPN	Seychelles	SYC
Botswana	BWA	Jordan	JOR	Sierra Leone	SLE
Brazil	BRA	Kazakhstan	KAZ	Singapore	SGP
Brunei Darussalam	BRN	Kenya	KEN	Slovakia	SVK
Bulgaria	BGR	Kiribati	KIR	Slovenia	SVN
Burkina Faso	BFA	Kuwait	KWT	Solomon Islands	SLB
Burundi	BDI	Kyrgyzstan	KGZ	Somalia	SOM
Cambodia	KHM	Lao PDR	LAO	South Africa	ZAF
Cameroon	CMR	Latvia	LVA	South Sudan	SDS
Canada	CAN	Lebanon	LBN	Spain	ESP
Cape Verde	CPV	Lesotho	LSO	Sri Lanka	LKA
Central African Republic	CAF	Liberia	LBR	Sudan	SDN
Chad	TCD	Libya	LBY	Suriname	SUR
Chile	CHL	Lithuania	LTU	Sweden	SWE
China	CHN	Luxembourg	LUX	Switzerland	CHE
Colombia	COL	Macao	MAC	Syria	SYR
Comoros	COM	Macedonia	MKD	Taiwan	B77
Costa Rica	CRI	Madagascar	MDG	Tajikistan	TJK
Côte d'Ivoire	CIV	Malawi	MWI	Tanzania	TZA
Croatia	HRV	Malaysia	MYS	Thailand	THA
Cuba	CUB	Maldives	MDV	The Gambia	GMB
Curaçao	CUW	Mali	MLI	Timor-Leste	TLS
Cyprus	CYP	Malta	MLT	Togo	TGO
Czech Republic	CZE	Mauritania	MRT	Tonga	TON
Dem. Rep. Korea	PRK	Mauritius	MUS	Trinidad and Tobago	TTO
Dem. Rep. of the Congo	COD	Mexico	MEX	Tunisia	TUN
Denmark	DNK	Moldova	MDA	Turkey	TUR
Djibouti	DJI	Mongolia	MNG	Turkmenistan	TKM
Dominican Republic	DOM	Montenegro	MNE	Uganda	UGA
Ecuador	ECU	Morocco	MAR	Ukraine	UKR
Egypt	EGY	Mozambique	MOZ	United Araba Emirates	ARE
El Salvador	SLV	Myanmar	MMR	United Kingdom	GBR
Equatorial Guinea	GNQ	Namibia	NAM	United States	USA
Eritrea	ERI	Nepal	NPL	United States Virgin Islands	VIR
Estonia	EST	Netherlands	NLD	Uruguay	URY
Ethiopia	ETH	New Caledonia	NCL	Uzbekistan	UZB
Fed. St. Micronesia	FSM	New Zealand	NZL	Vanuatu	VUT
Fiji	FJI	Nicaragua	NIC	Venezuela	VEN
Finland	FIN	Niger	NER	Vietnam	VNM
France	FRA	Nigeria	NGA	Western Sahara	B28
French Polynesia	PYF	Norway	NOR	Yemen	YEM
Gabon	GAB	Oman	OMN	Zambia	ZMB
Georgia	GEO	Pakistan	PAK	Zimbabwe	ZWE
Germany	DEU	Palestine	PSX		

Data source WPP = [1].

Reference

1. United Nations, Department of Economic and Social Affairs, Population Division. World population prospects 2019–Special agregates. 2023; Online Edition. Rev. 1. Available at `https://population.un.org/wpp/Download/SpecialAggregates/EconomicTrading/`, accessed 15 January 2023.
