# Supplementary material for: Convergence and divergence in mortality: A global study from 1990 to 2030
Source: PLoS One. 2024 Jan 17;19(1):e0295842. doi: 10.1371/journal.pone.0295842 (PMC10793939; doi:10.1371/journal.pone.0295842)
Supplement: S2 Annex — (PDF) [file pone.0295842.s002.pdf]

B Annex 2: Estimation of Mortality Indicators

In this annex, we showed how the mortality indicators employed in this paper are estimated.

1. Life expectancy, $e_{x,t}$ for individuals with age x and period t is expressed as:

$$e_{x,t} = \frac{T_{x,t}}{l_{x,t}} \tag{1}$$

where $T_{x,t}$ is the total number of expected years to live from age x to the maximum attainable age of the life table population in the year t and $l_{x,t}$ is the number of individuals aged x last birthday on January 1 of period t.

2. The Gini index, $G_{x_s,t}$ at age x_s is expressed as:

$$G_{x_s,t} = \frac{\sum_{x=x_s}^{\omega-1} \left(f_{x,t}^{x_s} - g_{x,t}^{x_s}\right)}{\sum_{x=x_s}^{\omega-1} f_{x,t}^{x_s}}, \tag{2}$$

where, $f_{x,t}^{x_s}$ is the probability of death between ages x_s and x,

$$f_{x,t}^{x_s} = 1 - \frac{l_{x,t}}{l_{x_s,t}} \tag{3}$$

and $g_{x,t}^{x_s}$ is the proportion of the total age lived by the individuals that have died at age x,

$$g_{x,t}^{x_s} = \frac{T_{x_s,t} - T_{x,t} - (x - x_s) \cdot l_{x_s,t}}{T_{x_s,t}}. \tag{4}$$

3. The conditional standard deviation, $s_{x_s,t}$ at age x_s has been proposed by [1] as:

$$s_{x_s,t} = \sqrt{\frac{\sum_{x=x_s}^{\omega} d_{x,t} \cdot (x + a_{x,t} - e_{x,t} - x_s)^2}{l_{x_0}}}, \tag{5}$$

where, $d_{x,t}$ is the number of deaths at the beginning of age x for a year t and $a_{x,t}$ is the average period of time lived with age x for those who die with age x (last birthday) during year t.

Reference

1. Edwards RD, Tuljapurkar S. Inequality in life spans and a new perspective on mortality convergence across industrialized countries. Population and Development Review. 2005; 31(4):645–674.
