# Supplementary material for: Convergence and divergence in mortality: A global study from 1990 to 2030
Source: PLoS One. 2024 Jan 17;19(1):e0295842. doi: 10.1371/journal.pone.0295842 (PMC10793939; doi:10.1371/journal.pone.0295842)
Supplement: S3 Annex — (PDF) [file pone.0295842.s003.pdf]

C Annex 3: Supplementary information on mortality indicators.

Table 2. Male cluster membership: list of countries by year.

Country	1990	2010	2030	Country	1990	2010	2030	Country	1990	2010	2030	Country	1990	2010	2030
ABW	3	4	4	DZA	3	3	2	LBR	1	1	1	RUS	4	4	4
AFG	1	1	1	ECU	2	3	3	LBY	3	4	4	RWA	5	1	4
AGO	1	1	1	EGY	3	4	4	LCA	2	3	3	B28	4	4	4
ALB	3	2	4	ERI	1	1	4	LKA	2	3	4	SAU	3	4	4
ARE	3	2	2	ESP	2	2	2	LSO	4	5	5	SDN	4	1	1
ARG	3	4	4	EST	3	4	2	LTU	3	4	4	SDS	1	1	1
ARM	3	4	4	ETH	1	1	4	LUX	2	2	2	SEN	4	1	1
ATG	2	3	2	FIN	2	2	2	LVA	3	4	4	SGP	2	2	2
AUS	2	2	2	FJI	4	4	4	MAC	2	2	2	SLB	3	4	4
AUT	2	2	2	FRA	2	2	2	MAR	3	4	4	SLE	1	1	1
AZE	4	4	4	FSM	4	1	1	MDA	3	4	4	SLV	3	3	3
BDI	1	1	1	GAB	4	1	1	MDG	1	1	4	SOM	1	1	1
BEL	2	2	2	GBR	2	2	2	MDV	4	4	2	SRB	3	4	4
BEN	4	1	1	GEO	3	4	4	MEX	2	3	3	STP	4	4	4
BFA	1	1	1	GHA	4	1	1	MKD	3	4	4	SUR	3	4	4
BGD	4	4	3	GIN	1	1	1	MLI	1	1	1	SVK	3	4	4
BGR	3	4	4	GMB	1	1	1	MLT	2	2	2	SVN	3	2	2
BHR	3	4	4	GNB	1	1	1	MMR	4	1	1	SWE	2	2	2
BHS	2	4	4	GNQ	1	1	1	MNE	2	4	4	SYC	3	4	4
BIH	3	4	4	GRC	2	2	2	MNG	4	4	4	SYR	3	4	4
BLR	4	4	1	GRD	3	4	4	MOZ	1	1	1	TCD	1	1	1
BLZ	2	3	4	GTM	3	3	3	MRT	4	1	1	TGO	4	1	1
BOL	4	3	3	GUM	3	2	2	MUS	3	4	4	THA	2	3	3
BRA	3	3	3	GUY	4	3	3	MWI	1	1	1	TJK	4	4	4
BRB	2	3	3	HKG	2	2	2	MYS	3	3	3	TKM	4	1	1
BRN	3	3	3	HND	2	3	3	NAM	4	1	1	TLS	1	4	4
BTN	4	3	3	HRV	3	4	4	NCL	3	4	4	TON	3	4	4
BWA	4	1	1	HTI	4	1	3	NER	1	1	1	TTO	3	3	3
CAF	1	1	5	HUN	3	4	4	NGA	1	1	1	TUN	3	4	4
CAN	2	2	2	IDN	4	4	4	NIC	3	3	3	TUR	4	4	2
CHE	2	2	2	IND	4	4	4	NLD	2	2	2	B77	2	3	3
CHL	2	3	2	IRL	3	2	2	NOR	2	2	2	TZA	1	1	4
CHN	3	4	4	IRN	3	4	4	NPL	4	4	4	UGA	5	1	1
CIV	1	1	5	IRQ	3	4	1	NZL	2	2	2	UKR	3	4	1
CMR	1	1	1	ISL	2	2	2	OMN	3	3	3	URY	3	3	4
COD	1	1	1	ISR	2	2	2	PAK	3	4	1	USA	2	2	2
COG	1	1	1	ITA	2	2	2	PAN	2	3	3	UZB	3	4	4
COL	2	3	3	JAM	2	3	4	PER	3	3	3	VCT	3	4	4
COM	4	1	1	JOR	3	4	4	PHL	3	4	4	VEN	2	3	4
CPV	3	4	4	JPN	2	2	2	PN1	4	1	1	VIR	2	2	2
CRI	2	3	3	KAZ	4	4	1	POL	3	3	3	VNM	2	3	3
CUB	2	3	3	KEN	4	1	1	PRI	2	3	2	VUT	4	4	4
CUW	2	3	3	KGZ	3	4	1	PRK	4	4	1	WSM	4	4	4
CYP	2	2	2	KHM	1	4	4	PR1	2	2	2	YEM	4	1	1
CZE	3	2	2	KIR	4	1	4	PRY	2	3	3	ZAF	4	1	1
DEU	2	2	2	KOR	3	2	2	PSX	3	4	4	ZMB	1	1	1
DJI	4	1	4	KWT	3	4	4	PYF	3	4	2	ZWE	1	1	5
DNK	2	2	2	LAO	4	1	4	QAT	2	3	3				
DOM	2	3	3	LBN	3	2	2	ROU	3	4	4				

Table 3. Female cluster membership: list of countries by year.

Country	1990	2010	2030	Country	1990	2010	2030	Country	1990	2010	2030	Country	1990	2010	2030
ABW	3	4	5	DZA	3	4	5	LBR	1	1	1	RUS 3	4	4	
AFG	1	1	4	ECU	2	3	3	LBY	3	4	4	RWA 5	1	4	
AGO	1	1	1	EGY	3	4	4	LCA	3	4	5	B28 4	4	4	
ALB	3	4	2	ERI	1	1	4	LKA	3	4	4	SAU 3	4	4	
ARE	3	4	5	ESP	2	2	2	LSO	4	5	1	SDN 4	1	4	
ARG	2	2	5	EST	3	2	2	LTU	2	2	5	SDS 1	1	1	
ARM	3	4	4	ETH	1	1	4	LUX	2	2	2	SEN 4	1	1	
ATG	3	4	5	FIN	2	2	2	LVA	3	3	5	SGP 2	2	2	
AUS	2	2	2	FJI	4	1	1	MAC	2	2	2	SLB 3	4	4	
AUT	2	2	2	FRA	2	2	2	MAR	3	4	4	SLE 1	1	1	
AZE	3	4	4	FSM	4	1	4	MDA	3	4	4	SLV 3	4	5	
BDI	1	1	1	GAB	4	1	1	MDG	1	1	4	SOM 1	1	1	
BEL	2	2	2	GBR	2	2	2	MDV	4	4	5	SRB 3	4	4	
BEN	4	1	1	GEO	3	4	4	MEX	2	3	5	STP 4	4	4	
BFA	1	1	1	GHA	4	1	1	MKD	3	4	4	SUR 3	4	4	
BGD	4	4	3	GIN	1	1	1	MLI	1	1	1	SVK 3	2	2	
BGR	3	4	4	GMB	1	1	1	MLT	2	2	2	SVN 2	2	2	
BHR	3	4	4	GNB	1	1	1	MMR	4	1	4	SWE 2	2	2	
BHS	2	4	4	GNQ	1	1	1	MNE	2	4	4	SYC 2	4	4	
BIH	3	4	5	GRC	2	2	2	MNG	4	4	4	SYR 3	4	4	
BLR	3	4	4	GRD	3	4	4	MOZ	1	1	1	TCD 1	1	1	
BLZ	3	4	4	GTM	3	4	5	MRT	4	1	1	TGO 4	1	1	
BOL	4	3	3	GUM	3	2	2	MUS	2	3	5	THA 2	3	5	
BRA	3	3	3	GUY	4	3	5	MWI	1	1	1	TJK 4	4	4	
BRB	2	3	5	HKG	2	2	3	MYS	3	4	5	TKM 3	4	4	
BRN	3	4	5	HND	3	3	5	NAM	4	1	1	TLS 1	1	4	
BTN	4	3	3	HRV	3	4	2	NCL	3	4	5	TON 4	4	4	
BWA	4	1	1	HTI	4	1	1	NER	1	1	1	TTO 3	4	5	
CAF	1	1	1	HUN	3	4	5	NGA	1	1	1	TUN 3	4	4	
CAN	2	2	2	IDN	4	4	5	NIC	3	3	5	TUR 3	4	2	
CHE	2	2	2	IND	4	4	4	NLD	2	2	2	B77 2	2	2	
CHL	2	3	5	IRL	2	2	2	NOR	2	2	2	TZA 1	1	4	
CHN	3	4	5	IRN	4	4	4	NPL	4	4	4	UGA 1	1	1	
CIV	1	1	1	IRQ	3	4	4	NZL	2	2	2	UKR 3	4	4	
CMR	1	1	1	ISL	2	2	2	OMN	3	3	3	URY 2	3	3	
COD	1	1	1	ISR	2	2	2	PAK	4	1	1	USA 2	2	5	
COG	4	1	1	ITA	2	2	2	PAN	2	3	3	UZB 3	4	4	
COL	2	3	5	JAM	3	4	4	PER	3	4	5	VCT 3	4	4	
COM	4	1	1	JOR	3	4	4	PHL	3	4	5	VEN 2	3	4	
CPV	4	4	4	JPN	2	2	2	PN1	4	1	1	VIR 2	2	2	
CRI	2	3	2	KAZ	3	4	4	POL	3	2	2	VNM 2	3	5	
CUB	2	3	5	KEN	4	1	1	PRI	2	3	2	VUT 4	4	4	
CUW	2	3	5	KGZ	3	4	4	PRK	3	4	4	WSM 3	4	4	
CYP	2	2	2	KHM	4	1	4	PR1	2	2	2	YEM 4	1	1	
CZE	3	2	2	KIR	4	4	5	PRY	3	4	5	ZAF 4	1	1	
DEU	2	2	2	KOR	2	2	2	PSX	3	4	4	ZMB 1	1	4	
DJI	4	1	1	KWT	3	4	4	PYF	3	4	5	ZWE 4	1	1	
DNK	2	2	2	LAO	4	1	4	QAT	5	3	3				
DOM	3	3	3	LBN	3	2	2	ROU	3	4	5				

Table 4. Characteristics of the 1990 clusters

MALE	MC1-1990			MC2-1990			MC3-1990			MC4-1990			MC5-1990		
Indicator	m	C	M	m	C	M	m	C	M	m	C	M	m	C	M
Representative country	Guinea-Bissau (31)			United States (54)			Trinidad and Tobago (64)			Yemen (43)			Rwanda, Uganda (2)		
$e_{0,t}$	37.11	44.91	51.94	64.95	72.23	76.25	60.19	66.50	72.60	52.62	56.58	65.81	20.38	30.85	41.33
$e_{65,t}$	10.02	11.13	12.38	14.08	15.35	17.19	11.77	13.16	14.31	8.91	11.47	13.35	9.80	10.62	11.45
M	70.00	70.00	75.00	70.00	80.00	80.00	70.00	75.00	80.00	65.00	70.00	75.00	25.00	32.50	40.00
$GI_{0,t}$	0.26	0.38	0.44	0.10	0.13	0.20	0.10	0.16	0.25	0.15	0.26	0.31	0.36	0.49	0.63
$GI_{65,t}$	0.05	0.05	0.05	0.05	0.06	0.08	0.05	0.05	0.06	0.04	0.05	0.06	0.05	0.05	0.05
$s_{0,t}$	24.56	30.85	33.35	15.02	17.99	25.50	15.30	20.92	29.10	18.76	27.75	31.11	26.24	26.57	26.90
$s_{65,t}$	6.07	6.56	7.03	7.42	8.18	9.14	6.72	7.52	8.24	5.43	6.93	7.83	6.26	6.74	7.23
$y_{50\%;0,t}$	36.58	50.29	61.01	69.30	73.97	77.32	62.91	69.34	73.21	54.09	63.94	69.11	3.47	22.64	41.81
$y_{75\%;65,t}$	67.67	68.63	69.57	70.12	71.67	73.44	68.50	69.92	71.40	67.33	68.90	70.45	67.48	67.94	68.39

FEMALE	FC1-1990			FC2-1990			FC3-1990			FC4-1990			FC5-1990		
Indicator	m	C	M	m	C	M	m	C	M	m	C	M	m	C	M
Representative country	Central African Republic (29)			Barbados (53)			St. Vincent & Grenadines (68)			Tajikistan (42)			Rwanda, Qatar (2)		
$e_{0,t}$	38.01	50.09	54.65	72.54	77.17	82.41	65.93	72.10	76.51	55.07	60.97	69.73	23.29	50.37	77.45
$e_{65,t}$	10.67	12.08	12.88	16.12	18.30	20.47	13.15	15.44	17.09	9.48	12.80	14.35	11.73	14.84	17.95
M	70.00	75.00	75.00	75.00	85.00	85.00	75.00	80.00	85.00	70.00	75.00	80.00	15.00	42.50	70.00
$GI_{0,t}$	0.29	0.33	0.44	0.08	0.11	0.16	0.09	0.13	0.19	0.13	0.23	0.29	0.14	0.37	0.61
$GI_{65,t}$	0.05	0.05	0.05	0.05	0.06	0.07	0.05	0.06	0.07	0.04	0.05	0.06	0.05	0.08	0.10
$s_{0,t}$	29.21	29.99	33.70	13.84	17.61	22.94	14.39	19.21	26.01	18.22	27.18	31.16	16.92	22.52	28.11
$s_{65,t}$	6.20	6.79	7.30	7.49	8.21	8.96	6.43	7.69	8.70	5.67	7.10	8.07	7.44	8.60	9.76
$y_{50\%;0,t}$	34.56	56.67	63.70	75.16	79.26	83.25	70.78	74.87	77.73	60.83	68.34	72.14	6.19	41.39	76.58
$y_{75\%;65,t}$	68.43	69.40	69.86	71.96	75.10	78.18	70.94	72.44	74.18	67.78	69.98	71.32	68.53	70.26	71.99

Number recorder in brackets represents the number of countries in each cluster and period.

Table 5. Characteristics of the 2010 clusters

MALE	MC1-2010			MC2-2010			MC3-2010			MC4-2010			MC5-2010		
Indicator	min	Cent	max	min	Cent	max	min	Cent	max	min	Cent	max	min	Cent	max
Representative Country.	Uganda (52)			Austria (37)			Thailand (38)			Grenada (66)			Lesotho (1)		
$e_{0,t}$	47.31	57.66	65.25	75.11	78.37	80.58	65.45	71.59	45.22	64.29	70.26	75.61	45.22	45.22	45.22
$e_{65,t}$	9.80	11.60	13.83	15.51	17.96	19.37	14.92	16.69	9.57	11.21	13.57	15.35	9.57	9.57	9.57
M	70.00	75.00	75.00	80.00	85.00	85.00	70.00	80.00	40.00	70.00	75.00	80.00	40.00	40.00	40.00
$GI_{0,t}$	0.14	0.23	0.32	0.09	0.10	0.12	0.11	0.15	0.28	0.09	0.13	0.20	0.28	0.28	0.28
$GI_{65,t}$	0.04	0.05	0.06	0.05	0.06	0.06	0.06	0.06	0.05	0.05	0.05	0.06	0.05	0.05	0.05
$s_{0,t}$	19.34	25.38	29.48	13.52	14.72	17.16	15.13	20.04	23.45	14.31	17.72	25.88	23.45	23.45	23.45
$s_{65,t}$	5.85	6.70	7.77	7.53	8.22	8.57	8.04	8.55	6.29	6.49	7.52	8.38	6.29	6.29	6.29
$y_{50\%;0,t}$	51.11	63.00	68.93	75.66	79.51	81.48	68.68	74.47	44.43	65.64	71.81	76.32	44.43	44.43	44.43
$y_{75\%;65,t}$	67.95	68.98	70.66	72.17	74.75	76.43	70.97	72.94	67.23	68.52	70.35	72.88	67.23	67.23	67.23

FEMALE	FC1-2010			FC2-2010			FC3-2010			FC4-2010			FC5-2010		
Indicator	min	Cent	max	min	Cent	max	min	Cent	max	min	Cent	max	min	Cent	max
Representative Country	Uganda (54)			Netherlands (41)			Vietnam (25)			Jamaica (73)			Lesotho (1)		
$e_{0,t}$	50.51	61.14	69.55	79.04	83.14	86.47	69.61	79.06	51.31	68.95	75.54	80.62	51.31	51.31	51.31
$e_{65,t}$	10.77	13.24	15.30	18.41	21.08	23.92	16.92	19.68	13.69	12.75	16.53	18.79	13.69	13.69	13.69
M	70.00	75.00	80.00	85.00	85.00	90.00	75.00	85.00	35.00	75.00	80.00	85.00	35.00	35.00	35.00
$GI_{0,t}$	0.13	0.22	0.31	0.08	0.08	0.11	0.10	0.12	0.28	0.08	0.11	0.17	0.28	0.28	0.28
$GI_{65,t}$	0.05	0.05	0.06	0.05	0.06	0.08	0.07	0.08	0.06	0.05	0.06	0.07	0.06	0.06	0.06
$s_{0,t}$	19.34	25.60	29.42	11.76	13.33	16.74	14.51	18.49	25.88	12.68	17.10	24.22	25.88	25.88	25.88
$s_{65,t}$	6.21	6.98	8.13	7.61	8.03	8.66	8.54	9.01	7.56	6.65	7.79	8.55	7.56	7.56	7.56
$y_{50\%;0,t}$	54.91	67.47	73.28	80.62	83.95	87.23	72.28	80.76	49.16	72.72	77.34	80.97	49.16	49.16	49.16
$y_{75\%;65,t}$	68.63	70.45	72.47	75.82	78.65	81.69	71.97	75.54	70.35	70.30	73.60	76.31	70.35	70.35	70.35

Number recorder in brackets represents the number of countries in each cluster and period.

Table 6. Characteristics of the 2030 clusters

MALE	MC1-2030			MC2-2030			MC3-2030			MC4-2030			MC5-2030		
Indicator	min	Cent	max	min	Cent	max	min	Cent	max	min	Cent	max	min	Cent	max
Representative Country	Republic of Congo (47)			Austria (44)			Brazil (31)			Indonesia (68)			Lesotho (4)		
$e_{0,t}$	50.51	61.14	69.55	79.04	83.14	86.47	69.61	79.06	51.31	68.95	75.54	80.62	51.31	51.31	51.31
$e_{65,t}$	10.77	13.24	15.30	18.41	21.08	23.92	16.92	19.68	13.69	12.75	16.53	18.79	13.69	13.69	13.69
M	70.00	75.00	80.00	85.00	85.00	90.00	75.00	85.00	35.00	75.00	80.00	85.00	35.00	35.00	35.00
$GI_{0,t}$	0.13	0.22	0.31	0.08	0.08	0.11	0.10	0.12	0.28	0.08	0.11	0.17	0.28	0.28	0.28
$GI_{65,t}$	0.05	0.05	0.06	0.05	0.06	0.08	0.07	0.08	0.06	0.05	0.06	0.07	0.06	0.06	0.06
$s_{0,t}$	19.34	25.60	29.42	11.76	13.33	16.74	14.51	18.49	25.88	12.68	17.10	24.22	25.88	25.88	25.88
$s_{65,t}$	6.21	6.98	8.13	7.61	8.03	8.66	8.54	9.01	7.56	6.65	7.79	8.55	7.56	7.56	7.56
$y_{50\%;0,t}$	54.91	67.47	73.28	80.62	83.95	87.23	72.28	80.76	49.16	72.72	77.34	80.97	49.16	49.16	49.16
$y_{75\%;65,t}$	68.63	70.45	72.47	75.82	78.65	81.69	71.97	75.54	70.35	70.30	73.60	76.31	70.35	70.35	70.35

FEMALE	FC1-2030			FC2-2030			FC3-2030			FC4-2030			FC5-2030		
Indicator	m	C	M	m	C	M	m	C	M	m	C	M	m	C	M
Representative Country	South Africa (42)			Ireland (42)			Brazil (11)			São Tomé and Principe (61)			El Salvador (38)		
$e_{0,t}$	49.30	62.82	70.64	82.21	86.54	90.24	77.63	83.62	90.74	70.69	76.65	80.81	72.20	80.78	85.40
$e_{65,t}$	11.32	13.87	17.88	19.73	23.64	26.84	20.33	22.76	27.27	13.20	16.42	18.63	18.37	20.71	23.05
M	75.00	80.00	80.00	85.00	90.00	95.00	80.00	85.00	95.00	75.00	80.00	85.00	75.00	85.00	90.00
$GI_{0,t}$	0.14	0.20	0.32	0.07	0.08	0.12	0.13	0.15	0.22	0.07	0.10	0.15	0.08	0.11	0.18
$GI_{65,t}$	0.05	0.05	0.08	0.05	0.07	0.11	0.11	0.12	0.17	0.04	0.06	0.07	0.06	0.07	0.10
$s_{0,t}$	19.47	24.02	28.88	10.07	12.22	14.43	11.19	15.70	20.17	11.42	15.74	21.94	11.18	16.56	22.95
$s_{65,t}$	6.35	7.13	8.82	7.04	7.87	8.70	7.90	9.19	10.06	6.25	7.76	9.06	7.88	8.77	9.35
$y_{50\%;0,t}$	53.04	68.25	75.94	82.56	86.96	90.71	78.96	84.79	91.17	73.23	78.06	81.72	75.51	82.69	85.79
$y_{75\%;65,t}$	69.08	70.99	73.92	78.02	81.55	85.16	74.69	78.59	84.77	70.60	73.80	78.15	73.87	77.43	79.77

Number recorder in brackets represents the number of countries in each cluster and period.

Table 7. Indicators of representative countries

MALE	1990					2010					2030				
	MC1	MC2	MC3	MC4	MC5	MC1	MC2	MC3	MC4	MC5	MC1	MC2	MC3	MC4	MC5
Indicator	GNB	USA	TTO	YEM	RWA-UGA	UGA	AUT	THA	GRD	LSO	COG	AUT	BRA	IDN	LSO
$e_{0,t}$	44.91	72.23	66.50	56.58	30.85	57.66	78.37	71.59	70.26	45.22	62.55	81.97	75.73	72.42	46.82
$e_{65,t}$	11.13	15.35	13.16	11.47	10.62	11.60	17.96	16.69	13.57	9.57	12.16	20.29	18.79	14.76	9.95
M	70	80	75	70	32.5	75	85	80	75	40	75	85	80	80	40
$GI_{0,t}$	0.38	0.13	0.16	0.26	0.49	0.23	0.10	0.15	0.13	0.28	0.19	0.09	0.15	0.12	0.29
$GI_{65,t}$	0.05	0.06	0.05	0.05	0.05	0.05	0.06	0.06	0.05	0.05	0.05	0.06	0.08	0.06	0.05
$s_{0,t}$	30.85	17.99	20.92	27.75	26.57	25.38	14.72	20.04	17.72	23.45	23.22	13.56	19.08	17.03	24.57
$s_{65,t}$	6.56	8.18	7.52	6.93	6.74	6.70	8.22	8.55	7.52	6.29	6.75	8.29	9.28	7.90	6.36
$y_{50\%;0,t}$	50.29	73.97	69.34	63.94	22.64	63.00	79.51	74.47	71.81	44.43	67.36	83.19	77.96	73.73	46.91
$y_{75\%;65,t}$	68.63	71.67	69.92	68.90	67.94	68.98	74.75	72.94	70.35	67.23	69.53	77.79	74.25	71.34	67.52

FEMALE	1990					2010					2030				
	FC1	FC2	FC3	FC4	FC5	FC1	FC2	FC3	FC4	FC5	FC1	FC2	FC3	FC4	FC5
Indicator	CAF	BRB	VCT	TJK	RWA-QAT	UGA	NLD	VNM	JAM	LSO	ZAF	IRL	BRA	STP	SLV
$e_{0,t}$	50.09	77.17	72.10	60.97	50.37	61.14	83.14	79.06	75.54	51.31	62.82	86.54	83.62	76.65	80.78
$e_{65,t}$	12.08	18.30	15.44	12.80	14.84	13.24	21.08	19.68	16.53	13.69	13.87	23.64	22.76	16.42	20.71
M	75	85	80	75	42.5	75	85	85	80	35	80	90	85	80	85
$GI_{0,t}$	0.33	0.11	0.13	0.23	0.37	0.22	0.08	0.12	0.11	0.28	0.20	0.08	0.15	0.10	0.11
$GI_{65,t}$	0.05	0.06	0.06	0.05	0.08	0.05	0.06	0.08	0.06	0.06	0.05	0.07	0.12	0.06	0.07
$s_{0,t}$	29.99	17.61	19.21	27.18	22.52	25.60	13.33	18.49	17.10	25.88	24.02	12.22	15.70	15.74	16.56
$s_{65,t}$	6.79	8.21	7.69	7.10	8.60	6.98	8.03	9.01	7.79	7.56	7.13	7.87	9.19	7.76	8.77
$y_{50\%;0,t}$	56.67	79.26	74.87	68.34	41.39	67.47	83.95	80.76	77.34	49.16	68.25	86.96	84.79	78.06	82.69
$y_{75\%;65,t}$	69.40	75.10	72.44	69.98	70.26	70.45	78.65	75.54	73.60	70.35	70.99	81.55	78.59	73.80	77.43

Table 8. β convergence analysis. Results from OLS regression of the rate of change in indicators on initial period logged levels. All countries

Period	1990-2010		2010-2030	
Gender	Male	Female	Male	Female
Coefficient	β	β	β	β
$e_{0,t}$	-0.4315***	-0.4128***	-0.0743***	-0.1136***
$e_{65,t}$	0.1644***	0.0527*	0.1797***	0.0962***
M_t	-0.7739***	-0.8629***	0.0251	-0.5560***
$GI_{0,t}$	-0.2238***	-0.2456***	-0.1020***	-0.1845***
$GI_{65,t}$	0.0548	0.1259*	0.3587***	0.6736***
$s_{0,t}$	-0.0869***	-0.0872***	-0.0379	-0.0472*
$s_{65,t}$	-0.0393	-0.1398***	0.0175	-0.0061
$y_{50\%;0,t}$	-0.8252***	-0.7855***	0.0142	-0.0493*
$y_{75\%;65,t}$	0.4096***	0.2274***	0.3961***	0.2094***

$*$ significant at .10 level $**$ at .05 level $***$ at .01 level.
