# Supplementary material for: Convergence and divergence in mortality: A global study from 1990 to 2030
Source: PLoS One. 2024 Jan 17;19(1):e0295842. doi: 10.1371/journal.pone.0295842 (PMC10793939; doi:10.1371/journal.pone.0295842)
Supplement: S4 Annex — (PDF) [file pone.0295842.s004.pdf]

D Annex 4: Brief summary of the stages of demographic transition.

In this annex, we have included a simple summary of the stages of epidemiological transition described by [1]:

- The Age of Pestilence and Famine: characterized by high fluctuating mortality rates attributed mainly to wars, epidemics, and pandemics, with a significant impact on children and women (especially in relation to pregnancy and childbirth).
- The Age of Receding Pandemics: transition-age defined by a huge reduction in mortality rates due to improvements in sanitation and living standards; resulting in a decline in epidemic-pandemic related deaths. The main cause of death is caused by the prevalence of chronic degenerative diseases among the middle-aged and elderly.
- The Age of Degenerative and Man-Made Diseases: characterized by a period of mortality equilibrium, in which the rate of mortality reduction is slower than in the previous stage, and heart disease, cancer, and stroke become the leading causes of death, affecting older age groups.
- The Age of the Conquest to Extend of Life: defined by declining mortality rates in both men and women in advanced ages. The main causes of death are degenerative diseases, which are becoming increasingly frequent among older populations.
- Age of Limits: a stage where the boundaries of human longevity are explored.

Additionally, it is worth noting that there are alternative theories on the stages of epidemiological transition proposed by researchers such as [2-4].

References

1. Robine JM. Redefining the stages of the epidemiological transition by a study of the dispersion of life spans: The case of France. Population: An English Selection. 2001; 13(1):173-193.
2. Rogers RG, Hackenberg R. Extending epidemiologic transition theory: a new stage. Social Biology. 1987; 34(3-4):234–243.
3. Omran AR. The epidemiologic transition theory revisited thirty years later. World Health Statistics Quarterly. 1984; 53 (2,3,4):99–119.
4. Jaacks, LM, Vandevijvere S, Pan A, McGowan CJ, Wallace C, Imamura F, Mozaffarian D, Swinburn B, Ezzati M. The obesity transition: stages of the global epidemic. The Lancet Diabetes & Endocrinology. 2019; 7(3):231–240.
