# Supplementary material for: Convergence and divergence in mortality: A global study from 1990 to 2030
Source: PLoS One. 2024 Jan 17;19(1):e0295842. doi: 10.1371/journal.pone.0295842 (PMC10793939; doi:10.1371/journal.pone.0295842)
Supplement: Attachment — Submitted filename: myrefer.pdf [file pone.0295842.s005.pdf]

Title : Convergence and Divergence in Mortality : A Global Study From 1990 to 2030

This article aims to update the literature on “convergence or divergence in mortality” with, in its abstract, references to Wilson 2001 and Mesle 2004, but it is not.

Both already published articles consider the evolution since a much older and longer period, namely for developed countries the end of the 18th century and they are not proposing mortality projections.

Moreover, they essentially only use the level of mortality, namely life expectancy, and are not interested at other functions which can be derived from a life table.

But, the previous authors are also looking at other sources of data which is not the case for the proposed article. Meslé et al. will look for data by major cause of death to explain the divergence, for example of Eastern countries.

The proposed article looks at only one source, not even discussing the quality of the data neither on how they are constructed.

For many countries, at least all African countries, estimating mortality is a huge issue and abridged life tables proposed by the UN are produced by “model life tables” inducing between ages a link which is specific to the model and not to the country (as it is the case for countries where deaths by age are known from vital statistics and corresponding population at risk by censuses).

The article is a good statistical exercise, using the latest published methods in demographic and statistical R packages but on a data set which has been built by the UN for a less ambitious aim.

It seems to us that the authors are seeking to compensate the lack of knowledge on demographic data sources by adding all the ”modern” indicators derived from a unique abridged life table.

Let us yet describe their work which could be of interest if it was limited to countries where mortality rates are not already a result of a model.

Their first and interesting step was to select a set of 9 indicators of mortality which can be all estimated from a unique abridged life table.

Two indicators refer to the average level of mortality, life expectancy at birth (whose variations are mainly due to infant mortality) but also life expectancy at age 65 for countries where mortality at young ages does notplays more of a role.

Authors are also interested in a second dimension of mortality such as the standard deviation of ages at death, or a recent indicator of rectangularization of survival, or even the Gini index which are all linked in a way which is interesting to analyze but only on countries where mortality by age are estimated independently.

It could also be of interest to study the link between these 9 indicators in various ”model life tables” but this study remains to be done.

The geographical coverage is very ambitious since the analysis covers 124 countries, that is to say almost the entire world apart from a few islands.

Let us discuss why African countries (and many other countries without vital statistics or accurate data) should be excluded.

Civil registration is non-existent or incomplete for most African states so that demographers have strived to find other data and other methods to estimate the level of mortality.

We then understand that it is very ambitious to be interested in the standard deviation of the age at death or in the rectangularization of the survival function when an estimation of the simple level is already problematic.

Demographers are even not enough ambitious to pretend that their estimates of life expectancy are accurate. They are more pragmatic and want to estimate if the mortality conditions are improving or deteriorating over time.

Thus, since the end of the 20th century, surveys on reproductive health have been imposed, not only in Africa, including DHS surveys which have proven to be of good quality. And these surveys, which most often only concern women of childbearing age, allow, by asking the woman questions about the number of people who have died among her siblings and the timing of these deaths, to estimate the mortality of her cohort, since birth.

By taking into account the estimated growth of the population (there are regularly censuses for that), it is then possible to estimate the level of this mortality from birth to each reproductive age.

It is also necessary to use standard mortality tables, such as those of Coale and Demeny for example, that is to say statistical models estimated from historical data for countries with civil status such as Sweden or France and hypothesize that African countries have and will have a drop in mortality quite similar to that of developed countries.

By repeating such a survey 10 years later, we can measure the progress made or the regression (in the case of an epidemic like HIV) but we can hardly be interested in the rectangularization of their survival function or even in the evolution of the standard deviation at death because it is a modeling result and not a reliable measure.

Even for the non African countries, it is mandatory for the authors to explain how accurate are the data. And for each of these countries, the population division of the United Nations publishes a short summary on the origin of the data. This document should be cited and countries should be selected.

The presentation of the results is also disappointing.

The statistical method used on these 3 tables (1990, 2010, 2030) (9 indicators for 194 countries) belongs to the multivariate statistical analysis.

As any reader of Plos-one knows its geography, it could have been more interesting to discover the geographical proximity from one or two principal planes than a terrestrial globe.

Also after the viewing of the globe, we are still ignorant on the gap between the clusters. When a country moves to another cluster, how big is the change. By not publishing the correlation circle we don't know how the indicators are linked, how many of those indicators are useless etc.

For resuming, the statistical analysis is a huge ”software” work, on too many countries. The article is hardly informative on the past situation of mortality in the world as well as the current one and even less on the future.

Concerning the form, there is no effort to make tables and graphs readable.

Table 1 have to be in alphabetical order (Algeria, Cambodia, Chad are difficult to find).

Tables 2 and 3 are not very useable not only because the abbreviations to 3 letters are unreadable but because the numbers are already presented in the geographical graph.

In tables 4, 5 and 6, don't use abbreviations (m C M R. C.). The R.C. representative country could be moved to the third row (and not the last).

Use longer abbreviations in table 7 : for example replace GNB by Guinea.B, TTO by Tr.&Tob. etc.

Replace Amat, J. (2017) link with `https://github.com/JoaquinAmatRodrigo/Estadistica-con-R/blob/master/PDF_format/37_Clustering_y_Heatmaps.pdf`

Possible Advice for the authors : J. Amat is also proposing to add PCA to a cluster analysis.

Indeed, the cluster analysis does not add much compared to an analysis of the first principal components except that it reinforces the visual appreciation which results from a projection on a plane and the group changes are sometimes misleading because they result from a threshold effect and can be marginal. We would therefore have liked a real principal components analysis which would have highlighted on the first axis this famous level of mortality with a spread of all countries and on the second and third axes, the other dimensions of mortality which are dispersion (at the same level of life expectancy). On the correlation circle, we could have assessed the usefulness or uselessness of certain indicators among the 9 chosen. It would also have been interesting to visualize the countries and the 5 clusters because this way the reader would have better understood whether the group changes are a threshold effect or a real exception.
