# Supplementary material for: Convergence and divergence in mortality: A global study from 1990 to 2030
Source: PLoS One. 2024 Jan 17;19(1):e0295842. doi: 10.1371/journal.pone.0295842 (PMC10793939; doi:10.1371/journal.pone.0295842)
Supplement: Attachment — Submitted filename: Reviewer2_PlosOne.pdf [file pone.0295842.s006.pdf]

Response to Reviewer 2 of PlOS ONE: "Convergence and Divergence in Mortality: A Global Study From 1990 to 2030"

D. Atance[1,*], M. M. Claramunt[2], X. Varea[2] and J.M. Aburto[3,4]
[1] Universidad de Alcalá, Madrid, Spain.
[2] Universitat de Barcelona, Barcelona, Spain.
[3] London School of Hygiene and Tropical Medicine, London, UK.
[4] University of Oxford, Oxford, UK
[*] Corresponding author, e-mail: david.atance@uah.es (D.Atance).
Manuscript ID: PONE-D-23-11934.

October 10, 2023

We sincerely thank the reviewer for his valuable feedback, which has allowed us to improve the quality of the paper. Below, he will find a list of the points raised in the reviewer's report, along with an explanation of how we have addressed each of them. Also, in the manuscript he will find in red color the changes and suggestions.

1. This article aims to update the literature on "convergence or divergence in mortality" with, in its abstract, references to Wilson (2001) and Vallin and Meslé (2004), but it is not.

2. Both already published articles consider the evolution since a much older and longer period, namely for developed countries the end of the 18th century and they are not proposing mortality projections.

3. Moreover, they essentially only use the level of mortality, namely life expectancy, and are not interested at other functions which can be derived from a life table.

4. But, the previous authors are also looking at other sources of data which is not the case for the proposed article. Meslé et al. (1998) will look for data by major cause of death to explain the divergence, for example of Eastern countries.

   Regarding these four points, we thank the reviewer for their thorough description of previous papers. These are acknowledged and cited in the manuscript and we agree with the reviewer on some points. It is true that the papers of Wilson (2001) and Vallin and Meslé (2004) focus primarily on the historical evolution of mortality trends, going back to the late 18th century, mainly in developed countries. As the reviewer notes, these papers do not incorporate mortality projections in their analyses and focus primarily on the metric of life expectancy.

   Our manuscript builds upon previous work described by the reviewer in multiple ways aiming at providing an update on the concept of mortality convergence/divergence. To this end, we conduct a comprehensive examination of nine different mortality indicators from a global perspective in recent decades and look forward. In contrast with previous

work, we use a more sophisticated statistical technique, namely clustering methods that consider both changes over time but also changes across different mortality indicators. In addition, a distinctive feature of this article is the exploration of mortality projections, providing insight into the prospective trajectories of convergence clubs. We have modified the abstract accordingly based on the reviewer's comments:

"*An empirical question that has motivated demographers is whether there is convergence or divergence in mortality/longevity around the world. The epidemiological transition is the starting point for studying a global process of mortality convergence. This manuscript aims to provide an update on the concept of mortality convergence/divergence. We perform a comprehensive examination of nine different mortality indicators from a global perspective using clustering methods in the period 1990-2030. In addition, we include analyses of projections to provide insights into prospective trajectories of convergence clubs, a dimension unexplored in previous work. The results indicate that mortality convergence clubs of 194 countries by sex resemble the configuration of continents. These five clubs show a common steady upward trend in longevity indicators, accompanied by a progressive reduction in disparities between sexes and between groups of countries. Furthermore, this paper shows insights into the historical evolution of the convergence clubs in the period 1990-2020 and expands their scope to include projections of their expected future evolution in 2030.*"

5. The proposed article looks at only one source, not even discussing the quality of the data neither on how they are constructed.

6. For many countries, at least all African countries, estimating mortality is a huge issue and abridged life tables proposed by the UN are produced by "model life tables" inducing between ages a link which is specific to the model and not to the country (as it is the case for countries where deaths by age are known from vital statistics and corresponding population at risk by censuses).

7. The article is a good statistical exercise, using the latest published methods in demographic and statistical R packages but on a data set which has been built by the UN for a less ambitious aim.

8. Let us yet describe their work which could be of interest if it was limited to countries where mortality rates are not already a result of a model.

9. The geographical coverage is very ambitious since the analysis covers 124 countries, that is to say almost the entire world apart from a few islands.

10. Let us discuss why African countries (and many other countries without vital statistics or accurate data) should be excluded.

11. Civil registration is non-existent or incomplete for most African states so that demographers have strived to find other data and other methods to estimate the level of mortality.

12. Even for the non African countries, it is mandatory for the authors to explain how accurate are the data. And for each of these countries, the population division of the United Nations publishes a short summary on the origin of the data. This document should be cited and countries should be selected.

Thanks to the reviewer for highlighting these important points. We would like to discuss several in deep comments:

- It should be emphasized that we rely on estimates provided by the United Nations Population Division (WPP). The WPP database seeks to produce reliable estimates of mortality and lifetables. Specifically, the WPP tries to incorporate the original mortality rates published by each country's national statistical institute from civil registries. However, in cases where a country lacks sufficient data on mortality rates, particularly in regions such as African and Asian countries, the WPP provides estimations using specific models. Consequently, it is evident that the quality of data varies between the countries within this database. Nevertheless, this fact does not justify the exclusion of some countries from our analysis. In fact, these life tables generated by WPP can still provide valuable data on mortality dynamics.
- Furthermore, we aim to provide a comprehensive overview of the WPP methodology regarding mortality data (United Nations, Department of Economic and Social Affairs, Population Division, 2022). Initially, WPP distinguished between child mortality (under five years of age) and overall mortality (ages above five years). Subsequently, in the process of estimating mortality rates estimates, the WPP attempts to use the original mortality rates from births, vital registrations, and deaths, provided by the national statistical institute of each country, a total of 113 countries. However, in populations where such data from vital registration are not available or of insufficient quality, for 124 countries, WPP uses a model life table to estimate mortality rates. Indeed, these models require one or more mortality indicators to estimate mortality rates that best describe the behavior of the mortality age pattern of a given country. Detailed information on countries that use registration data or model-specific life tables can be found in United Nations, Department of Economic and Social Affairs, Population Division (2022). In 2022, WPP uses five model life tables known as CD North, CD South, CD West, HIV/AIDS, and LogQuad models. For a deeper understanding of the construction of these model life tables, readers may refer to Li and Gerland (2011) and World Health Organization (2018). It is important to note that these five life table models are applied to estimate mortality rates in all African countries in the WPP database.
- It should be emphasized that the WPP methodology has improved over time the standards and quality of mortality estimates by using more advanced statistical techniques. Indeed, WPP has incorporated this year a Bayesian hierarchical model to estimate mortality rates for the probability of dying between the ages of 15 and 60 (Chao, 2020).

Thus, following the reviewer's recommendation, we have updated the first paragraph of Section 2 to add all previous points, as follows:

"*This study uses data by sex for the period 1990–2020 from United Nations, Department of Economic and Social Affairs, Population Division (2023). This database provides accessible demographic data for all countries and areas of the world. Except Channel Islands, Eswatini, French Guiana, Guadeloupe, Martinique, Mayotte and Réunion*[1]. *Various other sources could be employed such as Human Mortality Database (2020), we carry out an ambitious study around the world using the remaining 194 countries which contains mortality data from European countries, USA, Canada, Chile, Israel, Japan, New Zealand, South Korea and Taiwan, and Latin American Human Mortality Database Urdinola and Queiroz (2020) including information for Latin American countries*[2]. *However, to maintain consistency*

[1]Countries with a reduced population number, and most being islands.

[2]These two databases contain high-quality mortality information from the census and the death registry of the national statistical institute of each respective country.

across all the countries included in the study and to achieve large geographic coverage, even at the cost of using data of questionable quality, we decided to use a single database[3]*. Table 1 includes the list of the analyzed countries and their acronyms.*

It is essential to recognize that the United Nations Populations Division (WPP) seeks to generate reliable estimates of mortality and lifetables. Specifically, the WPP incorporates the original mortality rates published by each country's national statistical institute from births, vital registrations, and deaths. Nonetheless, in populations where such data from vital registration are not available or of insufficient quality, WPP uses model life tables for the estimation of mortality rates. In 2022, WPP employed five model life tables known as CD North, CD South, CD West, HIV/AIDS, and LogQuad models. For a deeper understanding of the construction of these five life tables, the reader may refer to Li and Gerland (2011) and World Health Organization (2018). Furthermore, it is worth emphasizing that the WPP methodology has evolved over time, incorporating new techniques. For instance, in 2022, Bayesian hierarchical (Chao, 2020) were integrated to estimate mortality rates between the ages of 15 and 60."

13. It seems to us that the authors are seeking to compensate the lack of knowledge on demographic data sources by adding all the "modern" indicators derived from a unique abridged life table.

    We appreciate the valuable feedback provided by the reviewer and would like to offer further clarification regarding the selection of mortality indicators, the use of abridged life tables, and the choice of data sources in our study.

    The decision to incorporate a set of nine mortality indicators seeks to provide an exhaustive analysis of global mortality convergence and divergence across all mortality perspectives over a specific period. To achieve this objective, we used the abridged life tables employed by the United Nations, Department of Economic and Social Affairs, Population Division (2022). It is worth noting that the use of these abridged life tables inherently implies that certain countries use model life tables or original data sources to construct these tables. Although it is recognized that other demographic data sources, such as Urdinola and Queiroz (2020) and Human Mortality Database (2020), provide life tables, these databases do not allow to cover the broad geographic coverage provided by the WPP database. One of the main contributions of the paper is the inclusion of almost the entire world to group the global mortality convergence and divergence.

    Thus to achieve a global study of mortality, from different perspectives, countries, regions, and periods, we decided to use these abridged life tables with the problems that this entails.

14. Their first and interesting step was to select a set of 9 indicators of mortality which can be all estimated from a unique abridged life table.

15. Two indicators refer to the average level of mortality, life expectancy at birth (whose variations are mainly due to infant mortality) but also life expectancy at age 65 for countries where mortality at young ages does notplays more of a role.

    We appreciate the reviewer's observation regarding to two mortality indicators used, such as the life expectancy at birth and life expectancy at age 65. Indeed, as the

[3] It should be noted that the analysis of convergence and divergence in mortality was initially done with three different databases. The clustering method is inclined to group countries in the same club/cluster from the same database. Therefore, to achieve greater consistency, we decided to use data from a single source.

reviewer highlights these two indicators capture different dimensions of mortality and offer valuable insights.

Life expectancy at birth is widely used in international comparison and provides a measure of the average length of life within a population. Indeed, this indicator is particularly influenced by variations in infant and child ages, making it a crucial indicator for assessing the health and well-being of a society, especially in regions with higher infant mortality rates. As such, it offers a comprehensive view of mortality conditions from the very beginning of life.

Life expectancy at age 65 measures the average expected lifetime among individuals who have already reached the age of 65. It is especially relevant for populations where mortality at young ages is relatively low, and most of deaths appear among older adults. By focusing on ages above 65, we minimize the extrinsic factor associated with infant mortality, related to high alcohol consumption, drug consumption, and smoking among younger ages (Thorslund et al., 2013). Additionally, this indicator provides insights associated with aging in a given society.

By including both indicators in our analysis, we aim to capture the different dimensions of the mortality phenomenon in the different age groups. While life expectancy at birth provides the impact of mortality in the infant and child ages, life expectancy at age 65 examines the mortality patterns among the elderly. Together, these two mortality indicators contribute to a more comprehensive assessment of mortality convergence and divergence across the world.

Thus, we have updated Section 3.1 to highlight the role played for both mortality indicators, life expectancy at birth and at age 65, as follows:

"*Life expectancy at x and time t, $e_{x,t}$ represents the average age at death if an individual experienced the prevailing death rates at time t throughout their lifetime from age x Debón et al. (2017); Aburto et al. (2018). It can be calculated for any age. We used $e_{0,t}$ and $e_{65,t}$ to denote life expectancy at birth and at age 65, respectively. By including both indicators in our analysis, we aim to capture the different dimensions of the mortality phenomenon in the different age groups. While $e_{0,t}$ provides the impact of mortality in the infant and child ages, $e_{65,t}$ examines the mortality patterns among the elderly and minimizes the extrinsic factor associated with infant mortality (Thorslund et al., 2013). Together, these two mortality indicators contribute to a more comprehensive assessment of mortality convergence and divergence across the world.*"

16. Authors are also interested in a second dimension of mortality such as the standard deviation of ages at death, or a recent indicator of rectangularization of survival, or even the Gini index which are all linked in a way which is interesting to analyze but only on countries where mortality by age are estimated independently.

17. We then understand that it is very ambitious to be interested in the standard deviation of the age at death or in the rectangularization of the survival function when an estimation of the simple level is already problematic.

    We appreciate the reviewer's observation regarding this point of our study. The inclusion of the standard deviation and the Gini index in our analysis contributes to a more global view of the mortality convergence/divergence phenomenon.

    It should be emphasized that the use of abbreviated life tables is not problematic because the results are consistent with previous studies related to high-income countries. Furthermore, in African countries, the results are in line with, we still obtain low

outcomes in life expectancy and also, high levels of standard deviation. Furthermore, WPP has improved the methodology over time incorporating more advanced statistical techniques, such as the Bayesian hierarchical model in 2022. Thus, it is evident that the quality of data varies between the countries within the WPP database. However, this fact does not justify the exclusion of some countries in our analysis.

In fact, we have introduced some changes in this regard in previous points, see the answer to 5-12 and 13. Additionally, we have incorporated the following paragraph at the conclusions:

"*It should be noted that our study uses abridged life tables from the United Nations, Department of Economic and Social Affairs, Population Division (2023) database. This is one of the weaknesses of the manuscript, we consider mortality data from WPP and hence, the mortality indicators can exhibit reduced accuracy, especially in African countries. This fact is due to these data being produced with model life tables. However, we decided to use this database for an ambitious study of mortality convergence and divergence covering a wide range of countries around the whole world. Therefore, future research may consider repeating this analysis for developed countries that have complete mortality data by age to get a really accurate study.*"

18. It could also be of interest to study the link between these 9 indicators in various "model life tables" but this study remains to be done.

    We appreciate this comment regarding how the mortality indicators were linked in various life tables. Thus, we have included an extra paragraph in the conclusions as a future line of research:

    "*Another future line of research, that we would like to explore, is to study the relationship between these nine indicators among the different life table models.*"

19. Demographers are even not enough ambitious to pretend that their estimates of life expectancy are accurate. They are more pragmatic and want to estimate if the mortality conditions are improving or deteriorating over time.

20. Thus, since the end of the 20th century, surveys on reproductive health have been imposed, not only in Africa, including DHS surveys which have proven to be of good quality. And these surveys, which most often only concern women of childbearing age, allow, by asking the woman questions about the number of people who have died among her siblings and the timing of these deaths, to estimate the mortality of her cohort, since birth.

21. By taking into account the estimated growth of the population (there are regularly censuses for that), it is then possible to estimate the level of this mortality from birth to each reproductive age.

    Thank you for your comments. We agree with the reviewer's observation that estimating mortality levels from birth to each reproductive age through surveys on reproductive health is indeed an interesting research question. However, we acknowledge that addressing these points falls beyond the scope of the current study. Nevertheless, we welcome these valuable suggestions as possible avenues for future research. we have included an additional paragraph at the end of the conclusion section:

    "*Another possible avenue for future research is to consider DHS surveys in the mortality estimates.*"

22. It is also necessary to use standard mortality tables, such as those of Coale and Demeny for example, that is to say statistical models estimated from historical data for countries with civil status such as Sweden or France and hypothesize that African countries have and will have a drop in mortality quite similar to that of developed countries.

23. By repeating such a survey 10 years later, we can measure the progress made or the regression (in the case of an epidemic like HIV) but we can hardly be interested in the rectangularization of their survival function or even in the evolution of the standard deviation at death because it is a modeling result and not a reliable measure.

    Thank you very much for the careful review. Indeed, we use projections from the Lee-Carter model (Lee and Carter, 1992) to forecast the mortality rates and, subsequently estimate mortality indicators for the period 2030. The Lee-Carter model has demonstrated its reliability as a predictive tool for forecasting mortality trends, as supported by various studies in the literature, including references such as Canudas-Romo et al. (2016); Atance et al. (2020) and Kessy et al. (2022).

    However, it is essential to acknowledge that these future estimations of the Lee-Carter model are based on past trends. These projections do not always accurately reflect future mortality trends, especially in the face of unexpected events such as wars, epidemics, or anomalous behavior. As the reviewer has rightly pointed out, an evaluation of our mortality estimates and cluster configurations in the year 2030 would be of considerable interest. Such an analysis would provide an assessment of the degree of accuracy of our 2023 estimates and the performance of the Lee-Carter model in projecting mortality trends and indicators.

    We appreciate their suggestion and, although this specific point is outside the scope of this paper, we recognize its potential importance as a topic for future research. Accordingly, we have included an additional paragraph in the Conclusions section to highlight this aspect:

    "*Study limitations should be mentioned. It is difficult to anticipate the future behavior of morality using a model based on past trends, which do not always accurately reflect the evolution of future mortality behavior. As a future line of research, it would be particularly interesting to review our mortality estimates and cluster configurations in 2030, when we will have reliable data. This future analysis would allow us to assess the degree of accuracy of our 2023 estimates and the performance of the Lee-Carter model in projecting mortality trends and indicators.*".

24. The presentation of the results is also disappointing.

    We have made some changes in order to improve the presentation of the results as follows:

    - We have added two figures (male and female) that present the geographical proximity of each country within each period based on the two principal components obtained through Principal Component Analysis (see reply to comment 26).
    - We have modified Table 1 to include the countries in alphabetical order (see reply to comment 29).
    - We have modified tables 2, 3, 4, 5, and 6 (see replies to comments 30 and 31).

25. The statistical method used on these 3 tables (1990, 2010, 2030) (9 indicators for 194 countries) belongs to the multivariate statistical analysis.

    We appreciate your comment on the statistical method employed in our study. Indeed, our paper uses a clustering method to study the mortality convergence and divergence covering 194 countries with nine mortality indicators in three time periods (1990, 2010, and 2030). Hence, this technique is considered a multivariate statistical analysis, since it is a statistical analysis of data containing observations in which more than one variable is measured.

    Thus, following this point, we have modified the first paragraph of section 4 (cluster methodology) to clarify that clustering methodology is considered a multivariate statistical analysis.

    "*We use a clustering method that has the purpose of finding patterns or subgroups (clusters) among a set of observations Hastie et al. (2009). This technique is considered a multivariate statistical analysis since it is a statistical analysis of data containing observations in which more than one variable is measured (Witten and James, 2013). The cluster methodology allows us to split the mortality indicators into different groups, so countries within each cluster are quite similar to each other, while countries in different clusters are quite different from each other. In this paper, we have grouped the countries according to the nine indicators explained in the previous section.*".

26. As any reader of PloS-One knows its geography, it could have been more interesting to discover the geographical proximity from one or two principal planes than a terrestrial globe.

    Thank the reviewer for this comment. As a response, we have included two additional paragraphs at the end of sections 5.1.1 and 5.1.2 where we describe the geographical proximity of the principal components derived from all the mortality indicators for the respective periods using the Principal Component Analysis (PCA), as follows:

    "*In addition, we have introduced Fig 1, which presents the geographical proximity of each country for males within each period based on the two principal components obtained through the Principal Component Analysis (PCA). This visualization allows us to depict the distance between the countries within each cluster for every period and, also, allows us to explain why countries move from one cluster to another in the next period, as a result of the distance to the next cluster. Notably, we observe a distinct pattern in MC5-1990, MC5-2010, and MC5-2030 where an independent cluster is grouped using two, one, and, four populations, respectively, with considerable distance from the remaining clusters.* "

Figure 1: **Geographical proximity between the two principal components in (a) 1990, (b) 2010, and (c) 2030 for the mortality indicators in males using PCA.**
The image was created using *factoextra* R-package, developed by Kassambara and Mundt (2020).

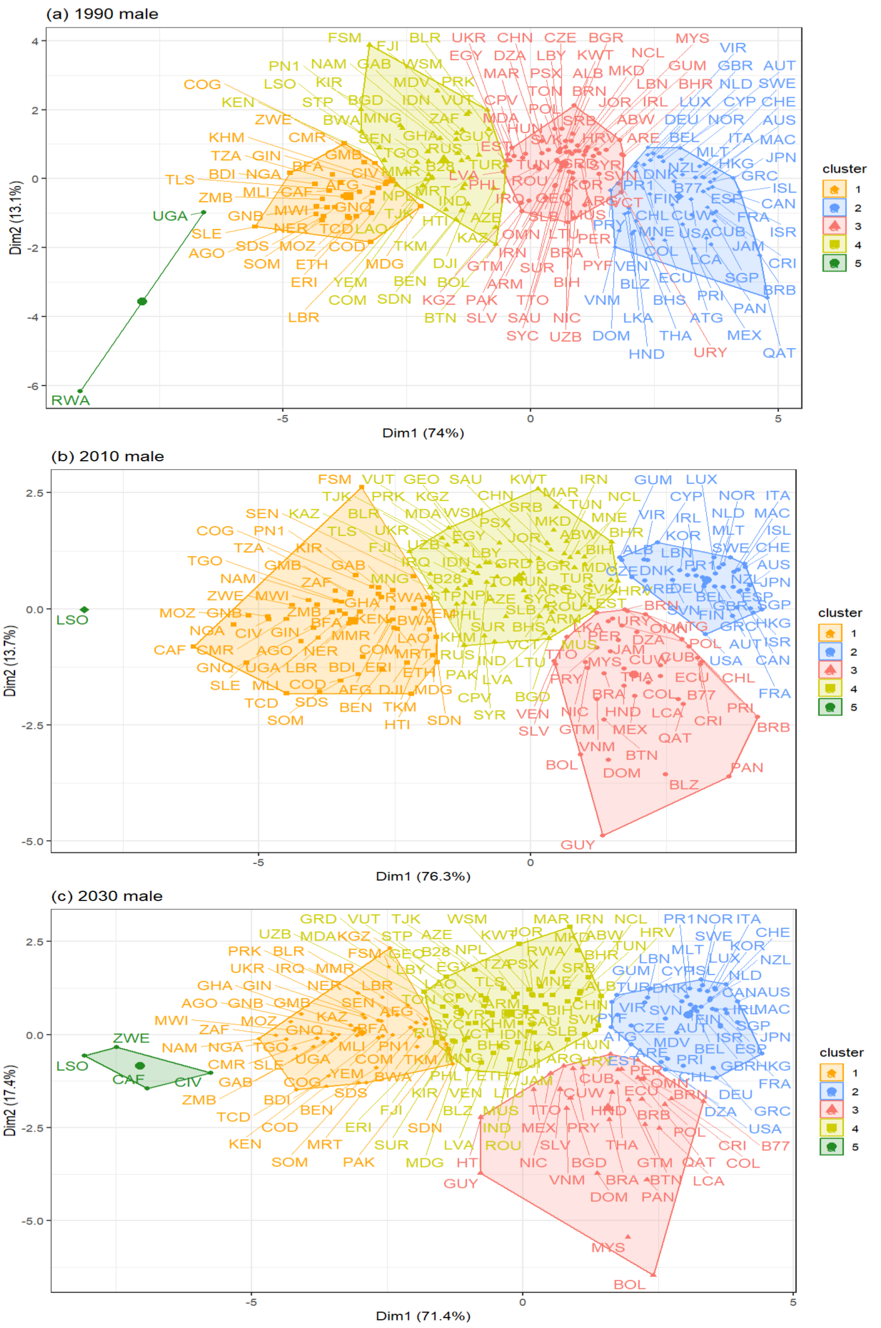

"Additionally, we have introduced Fig 2, which presents the geographical proximity of each country for females within each period based on the two principal components obtained through the Principal Component Analysis (PCA). Notably, we observe a distinct pattern in FC5-1990 and FC5-2010, where an independent cluster is grouped using two and one populations, respectively, with considerable distance from the remaining clusters. "

Figure 2: **Geographical proximity between the two principal components in (a) 1990, (b) 2010, and (c) 2030 for the mortality indicators in females using PCA.**
The image was created using *factoextra* R-package, developed by Kassambara and Mundt (2020).

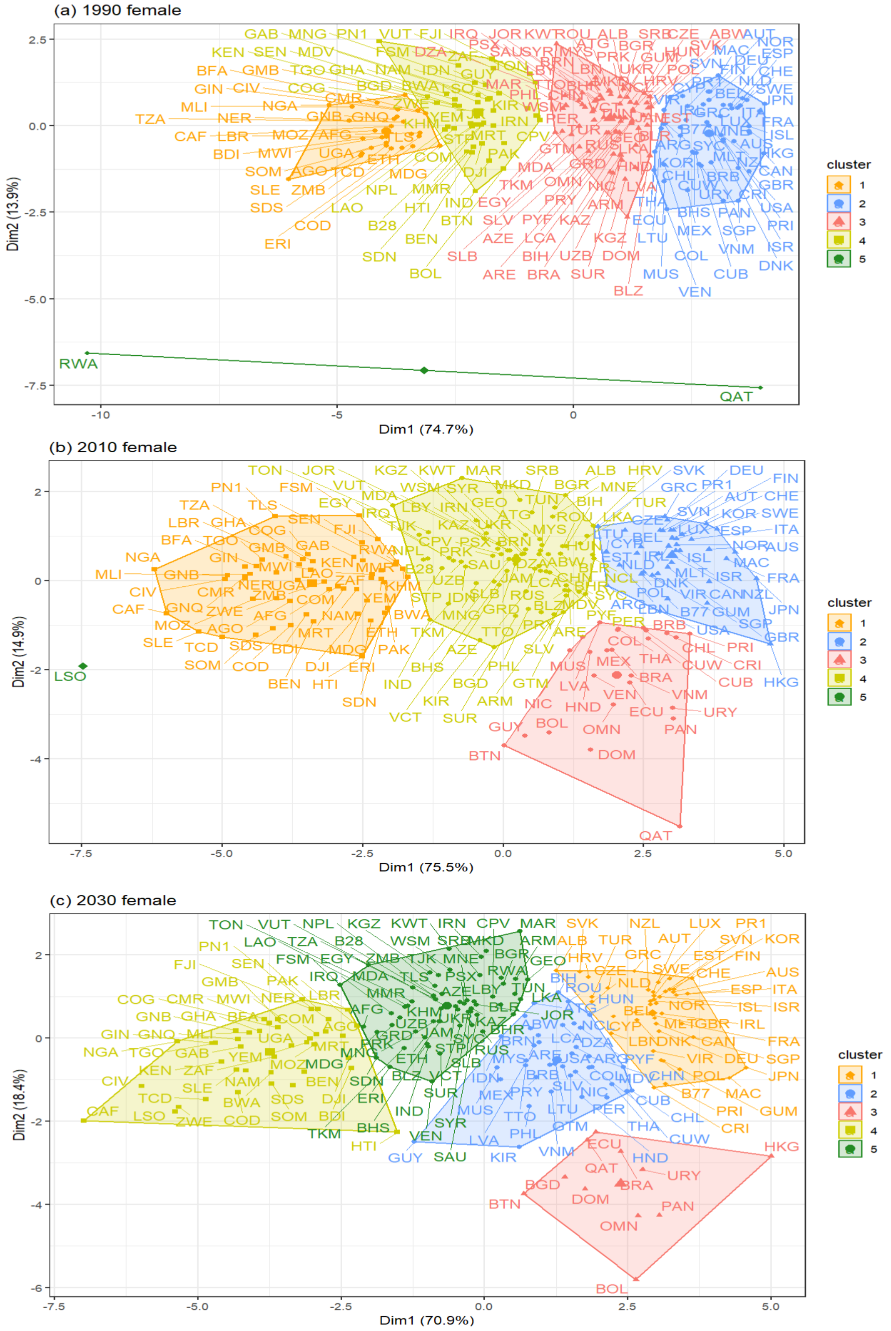

27. Also after the viewing of the globe, we are still ignorant on the gap between the clusters. When a country moves to another cluster, how big is the change. By not publishing the correlation circle we don't know how the indicators are linked, how many of those indicators are useless etc.

We appreciate the reviewer's comment regarding the correlations between the indicators. Thus, following your recommendation we have included an extra section, "3.6 Correlation between the mortality indicators". In this section, we have explained the correlation between the mortality indicators with a specific plot (Figure 3) and why we have included all of them in the study, as follows:

3.6 Correlation Between the mortality indicators

"*In this section, we analyze the correlation between the 9 mortality indicators ($e_{0,t}$, $e_{65,t}$, M_t, $G_{0,t}$, $G_{65,t}$, $s_{0,t}$, $s_{65,t}$, $y_{50\%;0,t}$, and $y_{75\%;65,t}$) used in this manuscript. Fig 3 presents the results. There are positive and highly (higher than 0.85) correlations between $e_{0,t}$, and $e_{65,t}$, $y_{50\%;0,t}$, and $y_{75\%;65,t}$. This outcome can be attributed to the fact that $e_{0,t}$ requires all the mortality rates for its estimation, while the other indicators use exclusively the end of the mortality curve. Consequently, countries with lower infant and young mortality rates tend to exhibit a high correlation between these indicators because the changes at the end of the mortality affect both indicators. Furthermore, $e_{0,t}$ is inversely and strongly (lower than -0.85) correlated with $G_{0,t}$ and $s_{0,t}$. This association arises because lower values of $G_{0,t}$ and $s_{0,t}$ indicate better results, while $e_{0,t}$ is the opposite, i.e., higher values indicate better results. The remaining correlations between the indicators can be observed in Fig Figure 3.*

We emphasize that all mortality indicators have been included in our study in order to cover different perspectives of the mortality phenomenon, despite the high positive and negative correlation between the mortality indicators. Indeed, when we performed a parallel analysis employing only $e_{0,t}$, M_t, $G_{0,t}$, and $G_{65,t}$, the clusters and number of countries in each cluster differed from the results presented in this manuscript. This fact underscores the value of incorporating a range of mortality indicators, as their inclusion leads to capturing the mortality phenomenon from all perspectives."

Figure 3: **Correlation plot between all mortality indicators used in the study, including both sexes and periods.**
The image was created using *corrplot* R-package, developed by Wei and Simko (2021).

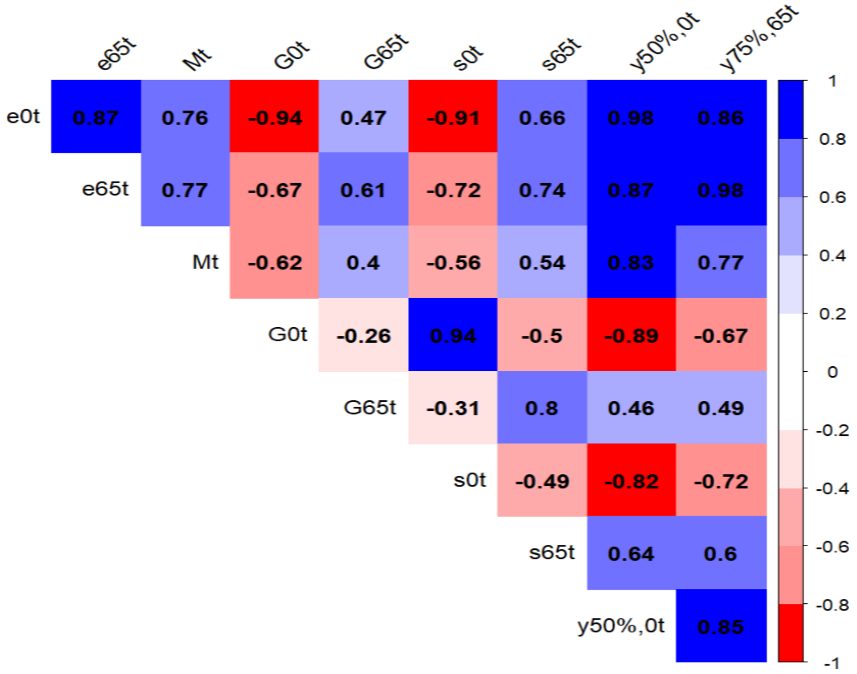

28. For resuming, the statistical analysis is a huge “software” work, on too many countries. The article is hardly informative on the past situation of mortality in the world as well as the current one and even less on the future.

29. Concerning the form, there is no effort to make tables and graphs readable. Table 1 have to be in alphabetical order (Algeria, Cambodia, Chad are difficult to find).

    Thank you for bringing this comment to our attention. Therefore, following the reviewer’s comment, we have modified Table 1 in alphabetical order for the country name.

30. Tables 2 and 3 are not very useable not only because the abbreviations to 3 letters are unreadable but because the numbers are already presented in the geographical graph.

    Following this comment, we have modified these tables in order to be more readable. It is true that they include abbreviations, but due to the size of the tables is not possible to include the full name of each country.

    Additionally, these tables are included in the manuscript to facilitate the cluster evolution of every country when a specific country can not be found in the geographical plot.

31. In tables 4, 5 and 6, don’t use abbreviations (m C M R. C.). The R.C. representative country could be moved to the third row (and not the last).

    Thanks for this comment. We have modified Tables 4, 5, and 6 in the manuscript to not use abbreviations and to modify the order of the rows.

32. Use longer abbreviations in table 7 : for example replace GNB by Guinea.B, TTO by Tr.& Tob. etc.

We appreciate this comment however the countries in Table 7 can not be changed by longer abbreviations due to the size of the table. However, we have improved all tables in order to facilitate the reading.

We appreciate this comment; however, due to space limitations, it is not feasible to replace country abbreviations with longer versions in Table 7. Nevertheless, we have made improvements to all tables to make them easier to read and understand.

33. Replace Amat (2017) link with `https://github.com/JoaquinAmatRodrigo/Estadistica-con-R/blob/master/PDF_format/37_Clustering_y_Heatmaps.pdf`.

    We apologize for this erroneous reference. We have modified the link for this cite as can be seen in the reference section of this file and in the manuscript (Amat, 2017).

34. Possible Advice for the authors : J. Amat is also proposing to add PCA to a cluster analysis.

35. Indeed, the cluster analysis does not add much compared to an analysis of the first principal components except that it reinforces the visual appreciation which results from a projection on a plane and the group changes are sometimes misleading because they result from a threshold effect and can be marginal. We would therefore have liked a real principal components analysis which would have highlighted on the first axis this famous level of mortality with a spread of all countries and on the second and third axes, the other dimensions of mortality which are dispersion (at the same level of life expectancy). On the correlation circle, we could have assessed the usefulness or uselessness of certain indicators among the 9 chosen. It would also have been interesting to visualize the countries and the 5 clusters because this way the reader would have better understood whether the group changes are a threshold effect or a real exception.

Finally, we would like to sincerely thank the reviewer for his careful review of the manuscript and valuable remarks.

References

Aburto, J. M., Wensink, M., van Raalte, A., and Lindahl-Jacobsen, R. (2018). Potential gains in life expectancy by reducing inequality of lifespans in Denmark: an international comparison and cause–of–death analysis. *BMC Public Health*, 18(1):1–9.

Amat, J. (2017). Clustering y heatmaps: aprendizaje no supervisado. Available at `https://github.com/JoaquinAmatRodrigo/Estadistica-con-R/blob/master/PDF_format/37_Clustering_y_Heatmaps.pdf` Accesed 14 January 2021.

Atance, D., Debón, A., and Navarro, E. (2020). A comparison of forecasting mortality models using resampling methods. *Mathematics*, 8(9):1550.

Canudas-Romo, V., DuGoff, E., Wu, A. W., Ahmed, S., and Anderson, G. (2016). Life expectancy in 2040: What do clinical experts expect? *North American Actuarial Journal*, 20(3):276–285.

Chao, F. (2020). Estimating age–sex–specific adult mortality and age–specific fertility rate in the World Population Prospects: A Bayesian modelling approach. UN Population Division, Technical Paper, No. forthcoming, New York, United Nations.

Debón, A., Chaves, L., Haberman, S., and Villa, F. (2017). Characterization of between–group inequality of longevity in European Union countries. *Insurance: Mathematics and Economics*, 75:151–165.

Hastie, T., Tibshirani, R., and Friedman, J. (2009). *The elements of statistical learning: data mining, inference, and prediction*. Springer Science & Business Media.

Human Mortality Database (2020). University of california, berkeley (usa), and max planck institute for demographic research (germany). *Avaliable at www. mortality. org and www. humanmortality. de. Accesed 1 January 2023.*

Kassambara, A. and Mundt, F. (2020). factoextra: Extract and visualize the results of multivariate data analyses. available at: `https://CRAN.R-project.org/package=factoextra`. R package version 1.0.7.

Kessy, S. R., Sherris, M., Villegas, A. M., and Ziveyi, J. (2022). Mortality forecasting using stacked regression ensembles. *Scandinavian Actuarial Journal*, 2022(7):591–626.

Lee, R. D. and Carter, L. R. (1992). Modeling and forecasting us mortality. *Journal of the American Statistical Association*, 87(419):659–671.

Li, N. and Gerland, P. (2011). Modifying the Lee–Carter method to project mortality changes up to 2100. In *Annual Meeting of the Population Association of America*.

Meslé, F., Vallin, J., and Shkolnikov, V. (1998). Reversal of mortality decline: the case of contemporary Russia. *World Health Statistics Quarterly*, 51(2-4):191–206.

Thorslund, M., Wastesson, J. W., Agahi, N., Lagergren, M., and Parker, M. G. (2013). The rise and fall of women's advantage: a comparison of national trends in life expectancy at age 65 years. *European Journal of Ageing*, 10:271–277.

United Nations, Department of Economic and Social Affairs, Population Division (2022). World Population Prospects 2022. Data Sources. UN DESA/POP/2022/DC/NO. 9. Available at `https://population.un.org/wpp/Download/Documentation/Documentation/` Accesed 14 September 2022.

United Nations, Department of Economic and Social Affairs, Population Division (2023). World population prospects 2019–Special agregates. *Online Edition. Rev. 1. Avaliable at https:// population. un. org/ wpp/ Download/ SpecialAggregates/ EconomicTrading/ Accesed 15 January 2023.*

Urdinola, B. and Queiroz, B. (2020). Latin American Human Mortality Database. *Avaliable at www. lamortalidad. org. Accesed 11 December 2020.*

Vallin, J. and Meslé, F. (2004). Convergences and divergences in mortality: a new approach of health transition. *Demographic Research*, 2:11–44.

Wei, T. and Simko, V. (2021). *R package 'corrplot': Visualization of a correlation matrix*. (Version 0.92).

Wilson, C. (2001). On the scale of global demographic convergence 1950–2000. *Population and Development Review*, 27(1):155–171.

Witten, D. and James, G. (2013). *An introduction to statistical learning with applications in R*. springer publication.

World Health Organization (2018). Global Health Estimates. Technical Paper WHO/HIS/IER/GHE/2018.2 Available at https://cdn.who.int/media/docs/default-source/gho-documents/global-health-estimates/lt_method_2016.pdf Accesed 10 December 2022.
